# Guidance for the treatment of adult growth hormone deficiency with somapacitan, a long-acting growth hormone preparation

**DOI:** 10.3389/fendo.2022.1040046

**Published:** 2022-12-23

**Authors:** Martin Bidlingmaier, Beverly M.K. Biller, David Clemmons, Jens Otto L. Jørgensen, Hiroshi Nishioka, Yutaka Takahashi

**Affiliations:** ^1^ Endocrine Laboratory, Medizinische Klinik und Poliklinik IV, Klinikum der Universität München, Munich, Germany; ^2^ Neuroendocrine & Pituitary Tumor Clinical Center, Massachusetts General Hospital and Harvard Medical School, Boston, MA, United States; ^3^ Department of Medicine, University of North Carolina, Chapel Hill, NC, United States; ^4^ Department of Endocrinology and Internal Medicine, Aarhus University Hospital, Aarhus, Denmark; ^5^ Department of Hypothalamic and Pituitary Surgery, Toranomon Hospital, Tokyo, Japan; ^6^ Department of Diabetes and Endocrinology, Nara Medical University, Kashihara, Japan; ^7^ Division of Diabetes and Endocrinology, Department of Internal Medicine, Kobe University Graduate School of Medicine, Kobe, Japan

**Keywords:** somapacitan, growth hormone, adult growth hormone deficiency, insulin-like growth factor I, treatment recommendations, pharmacokinetic/pharmacodynamic modelling, long-acting growth hormone

## Abstract

Adult growth hormone deficiency (AGHD) is a rare endocrine disorder characterized by an abnormal body composition, metabolic abnormalities associated with increased cardiovascular diseases, bone loss, and impaired quality of life. Daily subcutaneous injections with recombinant growth hormone (GH) can alleviate the abnormalities associated with AGHD. Several long-acting GH (LAGH) preparations are currently in development that aim to reduce treatment burden for patients receiving daily GH injections. Somapacitan (Sogroya^®^; Novo Nordisk, Denmark) is the first LAGH preparation that has been approved for treatment of AGHD in the United States, Europe, and Japan. The recent approval of somapacitan and anticipated approval of other LAGH molecules presents new questions for physicians planning to treat AGHD with LAGH in the future. Differences in the technologies used to prolong the half-life of recombinant GH are expected to result in variations in pharmacokinetic and pharmacodynamic profiles between preparations. Therefore, it is essential that physicians understand and consider such variations when treating patients with these novel GH replacement therapies. Here, we present a set of treatment recommendations that have been created to guide physicians initiating therapy with somapacitan in patients with AGHD who are eligible for GH replacement. Furthermore, we will review the published data that underlie these recommendations to explain the rationale for the treatment and monitoring advice provided.

## 1 Introduction

Adult growth hormone deficiency (AGHD), which can be caused by congenital abnormalities or acquired damage in the hypothalamic-pituitary region, is characterized by changes in body composition, decreased exercise capacity and quality of life (QoL), alterations in lipid profiles and other cardiovascular risk markers, and reductions in bone mass (1). Affected patients who receive daily subcutaneous injections with recombinant human growth hormone (GH) have improvement in the clinical features associated with AGHD (2, 3). While several well-established daily GH treatments have been in long-term clinical use, many patients are reluctant to initiate GH therapy due to the burden of daily injections. Since its initial approval by the US Food and Drug Administration in 1996 for the treatment of AGHD, recombinant GH replacement therapy has been administered to patients with AGHD *via* daily subcutaneous injections, which presents a barrier to treatment initiation and adherence (4). Several published studies have shown that treatment adherence is often poor for adult patients with GHD receiving daily GH treatment, contributing to suboptimal therapy outcomes (5–8). A long-acting GH (LAGH) preparation that requires less frequent dosing may therefore be beneficial for patients with AGHD. Several LAGH preparations are in clinical development for AGHD that use different technologies, including depot formulations, PEGylated molecules, pro-drug formulations, reversible albumin binding, and GH protein fusions (9–11). While daily recombinant GH products are uniform in structure, the methods used to prolong the action of GH can affect the molecule’s pharmacokinetic (PK) and pharmacodynamic (PD) properties (9). It is therefore essential for physicians to understand the PK-PD properties of these emerging products when using them clinically as differences may impact the initiation and monitoring of GH replacement therapy.

Somapacitan (Sogroya^®^; Novo Nordisk, Denmark) is a novel, albumin-binding GH preparation that was recently approved in the United States, Europe, and Japan for the treatment of AGHD. The safety and effectiveness of somapacitan for treatment of AGHD has been studied in three phase III clinical trials (ClinicalTrials.gov: REAL1 [NCT02229851], REAL2 [NCT02382939], REAL Japan [NCT03075644]) (12–14). The safety profile of somapacitan was shown to be comparable to that of daily GH, with no new safety issues being identified. In the REAL1 trial, the most frequent adverse events for both somapacitan and daily GH treatment groups (i.e., occurring in ≥5% of patients in any treatment arm) included upper respiratory tract infections, headache, back pain, arthralgia, gastroenteritis, and nasopharyngitis (13). Longer-term observational studies and post-marketing surveillance studies in a real-world setting are being initiated to provide further evidence to understand the long-term safety and effectiveness profile of somapacitan (i.e., global REAL7 study [planned] and Japanese post-marketing surveillance study [NCT05230550]).

As for all novel LAGH products, the switch from daily GH to somapacitan presents new practical questions for many endocrinologists planning to offer somapacitan therapy to their patients. This includes differences in clinical monitoring and dose titration, particularly regarding the appropriate insulin-like growth factor-I (IGF-I) target level and the timing of IGF-I sampling. Here, we present a set of treatment recommendations that have been developed to guide physicians initiating GH replacement therapy with somapacitan in newly diagnosed AGHD patients or patients currently treated with daily GH switching to somapacitan. The available data underlying these recommendations will also be reviewed to provide a rationale for clinical practice decisions.

## 2 Guidance for the use of somapacitan in AGHD

### 2.1 Development of treatment recommendations

Practical clinical recommendations for the treatment of AGHD with somapacitan were developed through a series of discussions with clinical experts, including but not limited to investigators who participated in clinical trials for somapacitan. The recommendations were drafted based on the authors’ clinical experience as well as information provided in approved product labels (United States, Europe, and Japan), published clinical and PK-PD modelling data (12–16), and relevant treatment guidelines (4, 17, 18). The participants contributing to development of the recommendations covered a wide geographic area and represented the regions where somapacitan is currently approved for treatment of AGHD. To initiate their development, a set of proposed treatment recommendations were first drafted based on the available data and discussed amongst the authors using a virtual discussion platform. Recommendations were revised based on the feedback gathered using the virtual discussion platform, and a virtual meeting was held in December 2021 to further discuss and refine the proposed recommendations and clinical practice considerations.

### 2.2 Treatment recommendations and clinical practice considerations

Based on the discussions, recommendations were created to guide physicians in the following areas:

- Initiating somapacitan in treatment-naïve patients (**Table 1**)
- Initiating somapacitan in patients switching from daily GH (**Table 2**).
- IGF-I-guided dose titration (**Table 3A**).
- Treatment evaluation (**Table 3B**).
- Missed doses (**Table 3C**).
- Special patient populations (**Table 4**).

**Table 1:** Recommendations for initiating GH replacement with somapacitan in treatment-naïve patients with AGHD.

	Recommendations	Clinical practice considerations
R1.1.	Treatment-naïve patients aged 18–59 years should receive a starting dose of 1.5 mg/week.	• Younger patients aged 18–30 years may require higher maintenance doses (4). In these patients, more frequent dose increases and/or larger dose increments may be needed.
R1.2.	Treatment-naïve patients aged 60 years and older should receive a starting dose of 1.0 mg/week.	• Men approaching 60 years of age may experience more adverse events (4, 17, 18). Physicians should therefore be more cautious with dose titration.
R1.3.	Treatment-naïve women on oral estrogen should receive a starting dose of 2.0 mg/week.	• Oral estrogen blunts hepatic production of IGF-I so that higher doses are needed in women taking oral estrogen (19–23). As women could receive oral estrogen for different reasons, including low-dose estrogen replacement therapy as part of a post-menopausal treatment plan, or higher-dose oral estrogen for females in their reproductive years, knowing whether a patient takes oral estrogen should be taken into account for GH dosing.
R1.4.	When initiating treatment with somapacitan, the treating physician should discuss with their patient and agree together on the injection day to ensure the feasibility of IGF-I sampling on day 3–4. Please also refer to R3.1.7.	• For example, if the patient would like to receive somapacitan injections on Wednesdays, and measurement of IGF-I is not possible during the weekend, the treating physician should encourage the patient to consider an earlier or later day in the week to facilitate testing during the appropriate window.

**Table 2:** Recommendations for somapacitan therapy in AGHD patients switching from daily GH to somapacitan.

	Recommendations	Clinical practice considerations
R2.1.	Patients aged 18–59 years switching from daily GH to somapacitan should receive a starting dose of 1.5 mg/week.	• According to the local product label in some countries (i.e., within the European Union), the starting dose for this group could be 2.0 mg/week. • Younger patients aged 18–30 years may require higher maintenance doses (4). In these patients, more frequent dose increases and/or larger dose increments may be needed.
R2.2.	Patients aged 60 years and older switching from daily GH to somapacitan should receive a starting dose of 1.0 mg/week.	• According to the local product label in some countries (i.e., within the European Union), the starting dose for this group could be 1.5 mg/week. • Men approaching 60 years of age may experience more adverse events (4, 17, 18). In these patients, more cautious dose titration may be needed.
R2.3.	Women on oral estrogen switching from daily GH to somapacitan should receive a starting dose of 2.0 mg/week.	• According to the local product label in some countries (i.e., within the European Union), the starting dose for this group could be 4.0 mg/week. • Oral estrogen blunts hepatic production of IGF-I so that higher doses are needed in women taking oral estrogen (19–23). As women receive oral estrogen for different reasons, including low-dose estrogen replacement therapy as part of a post-menopausal treatment plan, or higher-dose oral estrogen for females in their reproductive years, knowing whether a patient takes oral estrogen should be taken into account for GH dosing.
R2.4.	The first dose of somapacitan can be administered the day after the last dose of daily GH.	• A one-day washout period between the last dose of daily GH and first dose of somapacitan was used in clinical trials (14).
R2.5.	When patients are switched from daily GH to somapacitan, the treating physician should discuss with their patient and agree together on the injection day to ensure the feasibility of IGF-I sampling on day 3–4. Please also refer to **R3.1.7**.	• For example, if the patient would like to receive somapacitan injections on Wednesdays, and measurement of IGF-I is not possible during the weekend, the treating physician should encourage the patient to consider an earlier or later day in the week to facilitate testing during the appropriate window.

**Table 3(A):** Recommendations for IGF-I-guided dose titration and monitoring of AGHD patients treated with somapacitan.

	Recommendations	Clinical practice considerations
R3.1.1.	The somapacitan dose should be individually adjusted for each patient and increased gradually at 2–4-week intervals.	• Dose titration intervals should be spaced at least 2 weeks apart to allow the IGF-I response pattern to reach a steady state.
R3.1.2.	The dose should be titrated up or down at each step by 0.5 mg to 1.5 mg until the desired response is achieved.	
R3.1.3.	The dosage should be titrated based on clinical response, experience of adverse reactions, and guided by mean serum IGF-I concentrations.	• The evaluation of clinical response biomarkers such as body composition and quality of life are also important in addition to IGF-I monitoring.
R3.1.4.	The mean IGF-I SDS target for the week should aim to achieve the upper-normal range (0 to +2 SDS), not exceeding 2 SDS.	• The clinical practice guidelines recommend targeting the normal range (-2 to +2 SDS) (4). • The upper normal range is recommended in the European label, as this was targeted during clinical trials (12–14). • Published modelling data have indicated that there is a ratio of approximately 8.2 (observed interquartile range of 6.7–9.1) between somapacitan (mg/week) and somatropin (mg/day) maintenance doses that resulted in equivalent weekly IGF-I levels within the upper normal range (16). This conversion ratio therefore may be useful when estimating the required somapacitan maintenance dose for patients who have previously required very high or very low doses of daily GH. However, as this ratio represents the population mean derived from patients dosed in phase III trials, this conversion ratio may not be appropriate for all patients.
R3.1.5.	The somapacitan dose should be decreased or temporarily discontinued if patients experience adverse reactions or mean IGF-I concentrations for the week above the normal range.	
R3.1.6.	The maximum recommended dosage is 8 mg/week.	• This recommendation is based on this having been the highest dose tested in AGHD patients in clinical trials.
R3.1.7.	The mean IGF-I SDS for the week should be measured using a single sample drawn 3–4 days after the previous somapacitan dose.	• Based on modelling data, IGF-I levels measured on day 4 after dosing represent the best estimate of the weekly mean (15).
R3.1.8.	If measurement of IGF-I levels 3–4 days after the somapacitan dose is not feasible in a certain week, consider postponement of the measurement for one week and measure mean IGF-I levels 3–4 days after the next dose.	
R3.1.9.	If required, the dosing day can be changed provided that the interval between two doses is at least 4 days. After adjusting to a new dosing day, once-weekly dosing should be continued.	• PK-PD modelling data have shown that IGF-I levels are maintained if the somapacitan dose is delayed by 1–3 days (16).

**Table 3(B):** Recommendations for treatment evaluation.

	Recommendations	Clinical practice considerations
R3.2.1.	The treatment aim should be to achieve weekly mean IGF-I levels within the age-adjusted normal range within 12 months of beginning dose titration. If this target cannot be achieved, and the patient does not achieve the desired clinical response, treatment compliance should be assessed before alternate treatment options are considered.	
R3.2.2.	During somapacitan maintenance treatment, evaluation of efficacy and safety should be considered at approximately 6- to 12-month intervals. This includes evaluation of biochemistry (IGF-I, glucose, and lipid levels), body composition, and body mass index.	

**Table 3(C):** Recommendations for missed doses.

	Recommendations	Clinical practice considerations
R3.3.1.	A missed dose should be administered as soon as possible within the following 3 days. The regular once-weekly dosing schedule should then be resumed.	• PK-PD modelling data have shown that IGF-I levels are maintained if the somapacitan dose is delayed by 1–3 days (16).
R3.3.2.	If more than 3 days have passed since the missed dose, the dose should be skipped and the next dose administered on the regular dosing day.	

**Table 4:** Recommendations for special populations.

	Recommendations	Clinical practice considerations
R4.1.	Somapacitan should not be administered during pregnancy and in women of childbearing potential not using contraception.	• The evidence on safety of GH replacement therapy in this patient population is limited, which also is highlighted in current clinical practice guidelines (4).
R4.2.	When considering whether to continue or discontinue breast-feeding, the expected therapeutic benefits of somapacitan therapy for the mother and the benefits of breast-feeding for the child should be evaluated.	• Somapacitan is not recommended during breast-feeding due to lack of data.
R4.3.	Somapacitan should not be administered in patients with severe hepatic impairment (Child-Pugh score C). Patients with moderate hepatic impairment (Child-Pugh score B) should be treated with a starting dose of 1 mg/week. Dose titration should be performed based on individual requirements, with a maximum dose of 4 mg/week.	• Somapacitan exposure was shown to be significantly higher in subjects with moderate hepatic impairment (Child-Pugh score B) when compared with subjects with normal hepatic function (24). Subjects with severe hepatic impairment (Child-Pugh score C) were not included in this study.
R4.4.	Patients with renal impairment may require a lower starting dose of somapacitan (1 mg/week). Dose titration should be performed based on individual requirements.	• Somapacitan exposure was shown to be considerably higher in subjects with severe renal impairment (glomerular filtration rate (GFR) <30 mL/min) and those requiring hemodialysis when compared with subjects with normal renal function (GFR ≥90 mL/min) (24).
R4.5	Glucose levels should be monitored in all patients receiving somapacitan, especially for those with risk factors for diabetes mellitus (e.g., obesity, family history). Patients with type 1 or type 2 diabetes should be closely monitored and doses of antidiabetic drugs may need to be adjusted when patients initiate somapacitan.	• Treatment with GH can decrease insulin sensitivity, especially at higher doses (4, 17, 18). • Patients with undiagnosed diabetes or pre-diabetic patients may exhibit worsened glycemic control with GH treatment.

It is important to note that the provided recommendations are specific to somapacitan and based on clinical and PK-PD studies conducted in adults. These recommendations might therefore not be applicable for children or other LAGH products. In these cases, alternative recommendations may apply.

## 3 Evidence supporting recommendations and clinical practice considerations

Here, we review the published data that support the recommendations (**Tables 1**–**4**) and highlight differences between daily GH and somapacitan.

### 3.1 Starting doses for patients initiating therapy with somapacitan

Naïve patients or those switching from daily GH treatment to somapacitan should begin treatment using the starting doses defined in **Tables 1**, **2**. The starting dose differs according to age, with patients aged 18–59 years receiving 1.5 mg/week, and those aged ≥60 years receiving 1.0 mg/week. Women on oral estrogen treatment should receive a higher starting dose of 2.0 mg/week. The starting doses for these groups are included in the current product labels and have been selected based on those used in the REAL phase III clinical trials. These starting doses were shown to be safe and well-tolerated for the treatment of AGHD (12–14).

The use of differing starting doses in different age groups is based on data obtained from the clinical use of daily GH. Current guidelines state that lower doses should be administered for older patients as they are more responsive to GH, including its adverse effects (4, 17, 18). Considering the LAGH effect lasts longer than that of daily GH, it is important to avoid side effects of GH, including edema and arthralgia, especially when LAGH is initiated. Age-related changes in IGF-I secretion are known to occur over a person’s lifetime; after declining immediately after birth, IGF-I levels rise again until they reach a peak during puberty and decline gradually until old age (25). Younger adults, particularly those in the transition phase (i.e., up to 25–30 years of age), can potentially benefit from higher maintenance doses. IGF-I levels maintained at the upper end of the normal range can help to ensure that the effects of GH are optimized in these patients (26).

The recommendation to use higher somapacitan starting doses in women on oral estrogen is based on the ability for estrogen to blunt hepatic IGF-I production (19, 20). Oral estrogen replacement therapy can promote this effect by suppressing GH-dependent hepatic IGF-I production (21–23). In phase III trials, patients on oral estrogen received an average somapacitan maintenance dose of 3.8 mg/week, which is higher than the starting dose of 2.0 mg/week (16). This indicates that clinicians can anticipate that women on oral estrogen will need higher maintenance doses than women on no estrogen, or on transdermal estrogen (which does not have this effect).

Before initiating replacement therapy with GH, including LAGH, it should be noted that magnetic resonance imaging is recommended to evaluate the risk of tumor recurrence for patients with a history of pituitary tumors (4). If there is already tumor recurrence, but it is not found until after the patient has initiated GH replacement, GH could be incorrectly considered to be the cause for tumor growth. Active malignancy is included as a contraindication in the product label for somapacitan (27). There is currently no clear evidence that GH therapy causes growth of benign pituitary tumors (28, 29).

### 3.2 Somapacitan dose titration

#### 3.2.1 Introduction

After initiating patients on the starting doses described above, the dose should then be individualized gradually for each patient at 2–4-week intervals in steps of 0.5 mg to 1.5 mg (**Table 3A**). As with daily GH treatment, dose titration should be based on clinical response, experience of adverse reactions, and IGF-I levels.

#### 3.2.2 Use of IGF-I levels to guide dosing

Serum IGF-I levels can be used as a biomarker to evaluate adherence, safety, and efficacy during treatment with daily or weekly recombinant GH (4, 17, 18). IGF-I levels are also used to guide dosing for treatment-naïve patients and those switching to somapacitan (**Table 3A**). Differences between PD profiles for daily vs weekly GH must be observed for the meaningful interpretation of IGF-I concentrations (30). As detailed below, PK-PD modelling approaches have been used to predict how weekly IGF-I profiles are expected to differ for daily GH and somapacitan.

#### 3.2.3 Timing of IGF-I level measurement

**Figure 1:**
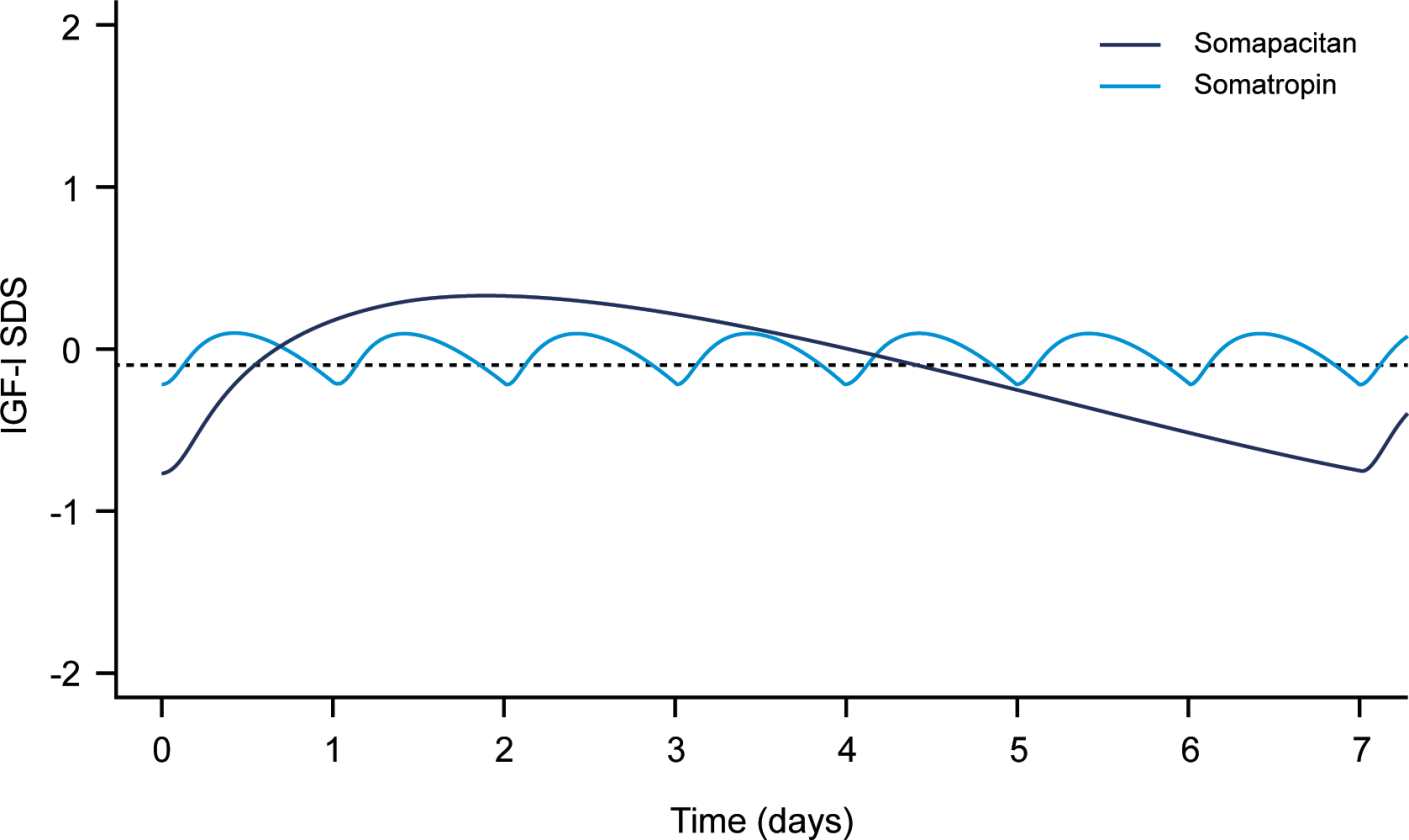
Simulated weekly IGF-I SDS levels following dosing with somapacitan (weekly GH [mean dose 2.4 mg]) and somatropin (daily GH [mean dose 0.3 mg]). Dashed line is weekly average IGF-I SDS for somapacitan (-0.09 SDS). Reproduced from Bentz Damholt et al. (24) under the CC BY-NC license (http://creativecommons.org/licenses/by-nc/4.0/).

IGF-I sampling should take place after the second or third weekly dose to ensure that steady state levels have been reached (15, 31). It is then recommended that IGF-I measurements be taken 3–4 days after administration of somapacitan (**Table 3A**) for the best estimate of weekly mean IGF-I levels as shown in two recently published modelling studies (15, 16). In the first study, a PK-PD model was used to simulate IGF-I levels following somapacitan dosing in children and adults using data derived from three phase I trials (NCT01514500; NCT01706783; NCT01973244) (15). In both adults and children, peak IGF-I levels were found to be best predicted from measurements taken on day two after administration and mean weekly IGF-I levels were best predicted from samples taken four days after dosing. In the second more recent study, somapacitan PK-PD models were based on data from phase III clinical trials in patients with AGHD (REAL1, REAL2, REAL Japan) (16). IGF-I profiles were simulated using the average maintenance doses for somapacitan and daily GH (somatropin). Model predictions have shown that daily GH treatment with a mean dose of 0.3 mg results in relatively stable IGF-I levels over a week, whereas treatment with somapacitan at a mean dose of 2.4 mg per week is expected to produce similar average IGF-I levels with a distinct weekly profile, peaking at two days after injection, reaching the mean value of the week around day 3–4, and reaching trough levels just before the next dose (**Figure 1**). It was concluded that IGF-I sampling should be performed on day 3–4 to calculate the weekly average. Mean weekly IGF-I levels measured three days after somapacitan dosing were used for dose titration in clinical trials, resulting in similar levels of effectiveness and safety when compared with daily GH (12–14). In addition, somapacitan treatment in these trials also achieved similar mean IGF-I levels as with daily GH (12–14).

#### 3.2.4 IGF-I target selection

Clinical practice guidelines recommend adjustment of dosing to target IGF-I levels within the age-adjusted reference range (-2 to +2 standard deviation score [SDS]) (4). The upper-normal range is recommended in the European product label for somapacitan as it was targeted during clinical trials (12–14) (**Table 3A**). Targeting IGF-I levels of -0.5 to +1.75 SDS in treatment-naïve AGHD patients in REAL1 was not associated with any safety signals compared to placebo for neither somapacitan nor daily GH (13). For patients from REAL2 switching from daily GH to somapacitan and targeting an IGF-I SDS of 0 to +2, no safety signals were identified for somapacitan or daily GH (12). Three studies from van Bunderen et al. have compared the effects of targeting the upper vs lower normal IGF-I range. In a randomized study comparing low-normal (−2 to −1 SDS) and high-normal (1 to 2 SDS) IGF-I target levels in AGHD, adults on daily GH targeting the high-normal range showed decreased waist circumference and improved QoL when compared with the low-normal group (32). However, higher IGF-I levels were associated with more myalgia. Another study examined memory and wellbeing in low vs high normal IGF-I groups (33). For female patients, high-normal IGF-I target levels were associated with reduced prefrontal cognitive functioning, and low-normal levels were associated with decreased vigor. A third study also showed that the high-normal range was associated with favorable effects on waist circumference, but negative effects on insulin resistance (34).

#### 3.2.5 Expected maintenance dose for patients switching from daily GH to somapacitan

Modelling analysis conducted using data from 330 patients with AGHD who received somapacitan in three phase III clinical trials indicated that there is an average ratio of 8.2 (interquartile range: 6.7–9.1) between somapacitan (mg/week) and somatropin (mg/day) maintenance doses that result in equivalent weekly IGF-I levels within the upper normal range (16). For example, a patient >60 years of age taking daily GH at a maintenance dose of 0.17 mg/day would be expected to receive 1.4 mg/week of somapacitan after dose titration. However, it is important to note that this conversion ratio was calculated as an average from all 330 AGHD patients, which included 70 patients >60 years, 188 patients ≤60 years, and 72 females on oral estrogen. Therefore, this ratio might not be appropriate for all patients, and it does not eliminate the need for individual dose titration. However, it may provide an estimate of the expected maintenance dose and could help the treating physician to adjust the dose titration plan accordingly.

The European Union has approved higher starting doses for patients switching from daily GH than for treatment-naïve patients. Higher doses were approved considering the observed average maintenance dose in somapacitan clinical trials (12–14). In the United States and Japan, the approved starting dose for patients switching to somapacitan from daily GH is the same as for treatment-naïve patients, and the products labels from both countries still recommend titrating the dose according to treatment response and IGF-I levels.

### 33 Missed doses of somapacitan

**Figure 2:**
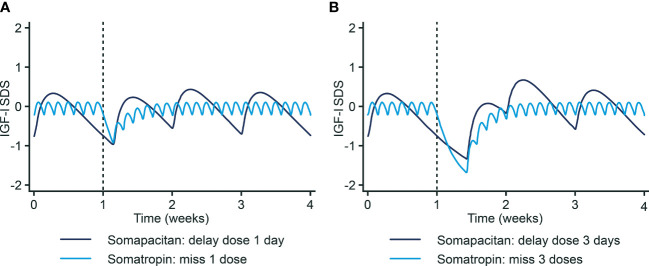
IGF-I SDS profiles following missed doses of somapacitan and somatropin. **(A)** One missed dose day. **(B)** Three missed dose days. Reprinted from Juul Kildemoes et al. (16) under the CC-BY license (https://creativecommons.org/licenses/by/4.0/). IGF-I, Insulin-like growth factor I. SDS, Standard deviation score.

If a dose of somapacitan is missed, it can be administered any time within the next three days. If more than three days have passed, the dose should be skipped (**Table 3C**). Recently published modelling data have shown that IGF-I levels are maintained if the somapacitan dose is delayed by 1–3 days (16). IGF-I SDS profiles for somapacitan (weekly) and somatropin (daily) GH after one and three missed dosing days are shown in **Figure 2**. Delaying the dose of somapacitan by one day had a negligible effect on the weekly IGF-I profile, whereas a delay of one day for somatropin required 3–4 days of subsequent dosing to restore IGF-I levels. Delaying the somapacitan dose by three days had a larger effect, but weekly IGF-I levels were largely maintained at appropriate levels. This may be useful information for patients who travel for work; they may not need to bring their medication and injections supplies if they will only be away for less than three days when their weekly injection is due.

### 3.4 Special patient populations receiving somapacitan

Use during pregnancy or in women of childbearing potential not currently using contraception should be avoided (**Table 4**). This is due to limited evidence in this patient population, which also is highlighted in current clinical practice guidelines (4).

**Figure 3:**
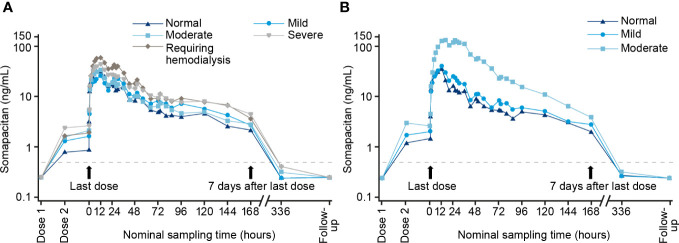
Geometric mean profile for somapacitan in patients with impaired kidney/liver function. **(A)** Normal function group and four kidney impairment groups. **(B)** Normal function group and two hepatic impairment groups (mild/moderate). Reproduced with permission (24).

Patients with renal/hepatic impairment are advised to receive lower starting doses of somapacitan (1 mg/week) (**Table 4**). Recombinant GH is cleared by both the kidneys and the liver (35, 36), so impaired kidney/liver function may alter the PK-PD properties of somapacitan. Recent data have indeed shown that somapacitan exposure is higher in adults with impaired kidney or liver function (24). As shown in **Figure 3**, there was a higher geometric mean concentration of somapacitan in groups with impaired kidney/liver function when compared with patients with normal function. Evaluation of the primary endpoint (area under the somapacitan serum concentration–time curve from time 0 to 168 hours after the last dosing [AUC_0–168 h_]) showed that somapacitan exposure was considerably higher in two of the kidney-impairment groups (‘severe’ and ‘requiring hemodialysis’) when compared with the normal function group. The AUC_0–168 h_ was significantly higher in patients with moderate liver impairment versus the group with normal function. Interestingly, it was observed that liver impairment appears to affect somapacitan clearance more than kidney impairment. It has been suggested that somapacitan is eliminated primarily by receptor-mediated clearance *via* GH receptors that are highly abundant in the liver (24). As patients with impaired kidney function included in this study had normal liver function, somapacitan could be cleared by the liver to a normal extent for this group, which support this observation. Overall, these data therefore need to be taken into account when making clinical decisions regarding use of somapacitan in patients with impaired kidney/liver function.

Blood glucose levels should be monitored regularly in all patients treated with somapacitan, especially those with risk factors for development of diabetes mellitus (e.g., obesity or family history of diabetes). Patients with type 1 or type 2 diabetes mellitus should be closely monitored, and doses of any antidiabetic medications may need to be adjusted when initiating treatment with somapacitan (**Table 4**). This is because daily (and potentially weekly) GH treatment may decrease insulin sensitivity, especially at higher doses (4, 17, 18). Patients with undiagnosed diabetes mellitus or pre-diabetes may experience worsened glycemic control during GH treatment. No clinically-relevant adverse effects on glucose metabolism were observed in naïve or previously treated AGHD patients during the phase III REAL clinical trials with somapacitan (37).

## 5 Conclusions

Somapacitan will offer patients the choice to administer GH therapy once weekly, reducing treatment burden when compared with daily treatment. These recommendations therefore provide simple and practical guidance to endocrinologists planning to offer somapacitan as GH replacement therapy in newly diagnosed AGHD patients or to their AGHD patients currently taking daily GH.

## Author contributions

All authors contributed to the article and approved the submitted version.
