# Corrigendum: Guidance for the treatment of adult growth hormone deficiency with somapacitan, a long-acting growth hormone preparation

**DOI:** 10.3389/fendo.2023.1158214

**Published:** 2023-03-02

**Authors:** Martin Bidlingmaier, Beverly M.K. Biller, David Clemmons, Jens Otto L. Jørgensen, Hiroshi Nishioka, Yutaka Takahashi

**Affiliations:** ^1^ Endocrine Laboratory, Medizinische Klinik und Poliklinik IV, Klinikum der Universität München, Munich, Germany; ^2^ Neuroendocrine & Pituitary Tumor Clinical Center, Massachusetts General Hospital and Harvard Medical School, Boston, MA, United States; ^3^ Department of Medicine, University of North Carolina, Chapel Hill, NC, United States; ^4^ Department of Endocrinology and Internal Medicine, Aarhus University Hospital, Aarhus, Denmark; ^5^ Department of Hypothalamic and Pituitary Surgery, Toranomon Hospital, Tokyo, Japan; ^6^ Department of Diabetes and Endocrinology, Nara Medical University, Kashihara, Japan; ^7^ Division of Diabetes and Endocrinology, Department of Internal Medicine, Kobe University Graduate School of Medicine, Kobe, Japan

**Keywords:** somapacitan, growth hormone, adult growth hormone deficiency, insulin-like growth factor I, treatment recommendations, pharmacokinetic/pharmacodynamic modelling, long-acting growth hormone

**Incorrect copyright statement**

In the published article, there was an error in the Copyright statement of **Figure 1**. The statement was incorrectly written as “Reproduced with permission (24).”. The corrected statement is “Reproduced from Bentz Damholt et al. (24) under the CC BY-NC license (http://creativecommons.org/licenses/by-nc/4.0/). No changes were made to this figure.”

**Figure 1:**
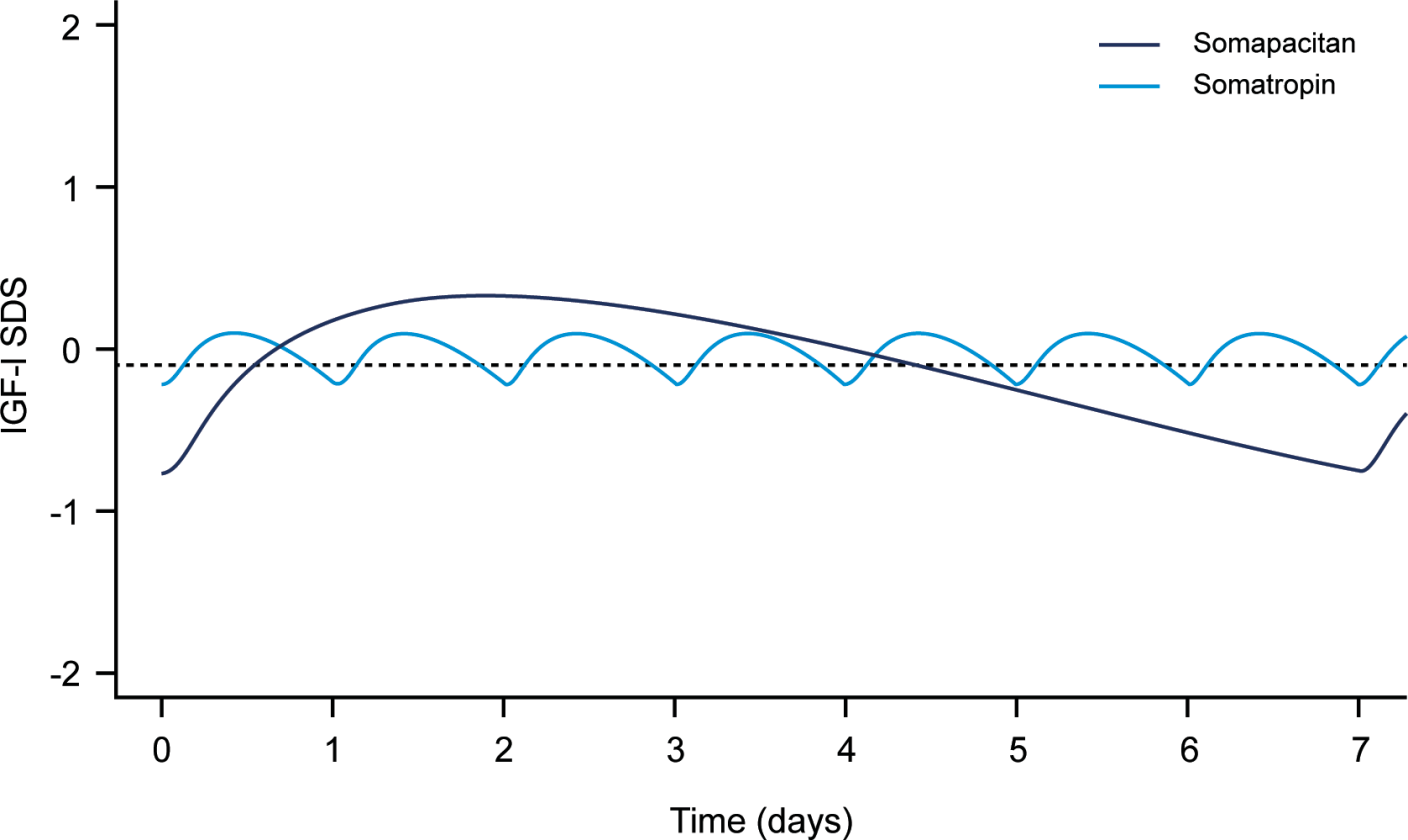
Simulated weekly IGF-I SDS levels following dosing with somapacitan (weekly GH [mean dose 2.4 mg]) and somatropin (daily GH [mean dose 0.3 mg]). Dashed line is weekly average IGF-I SDS for somapacitan (-0.09 SDS). Reproduced from Bentz Damholt et al. (24) under the CC BY-NC license (http://creativecommons.org/licenses/by-nc/4.0/).

The authors apologize for this error and state that this does not change the scientific conclusions of the article in any way. The original article has been updated.

